# Similarity and Differences in Inflammation-Related Characteristics of the Peripheral Immune System of Patients with Parkinson’s and Alzheimer’s Diseases

**DOI:** 10.3390/ijms18122633

**Published:** 2017-12-06

**Authors:** Anna A. Boyko, Natalya I. Troyanova, Elena I. Kovalenko, Alexander M. Sapozhnikov

**Affiliations:** Laboratory of Cell Interactions, Department of Immunology, Shemyakin–Ovchinnikov Institute of Bioorganic Chemistry, Moscow 117997, Russia; boyko_anna@mail.ru (A.A.B.); troyanatali@gmail.com (N.I.T.); lenkovalen@mail.ru (E.I.K.)

**Keywords:** Parkinson’s disease, Alzheimer’s disease, inflammation, neuroinflammation, peripheral immune system, cytokines, oxidative stress

## Abstract

Parkinson’s disease (PD) and Alzheimer’s disease (AD) are the most common age-related neurodegenerative disorders. Both diseases are characterized by chronic inflammation in the brain—neuroinflammation. The first signs of PD and AD are most often manifested in old age, in which the immune system is usually characterized by chronic inflammation, so-called “inflammaging” In recent years, there is growing evidence that pathogenesis of these diseases is connected with both regional and peripheral immune processes. Currently, the association of clinical signs of PD and AD with different characteristics of patient immune status is actively being researched. In this mini-review we compare the association of PD and AD alterations of a number of immune system parameters connected with the process of inflammation.

## 1. Introduction

The most common neurodegenerative diseases in the world are Alzheimer’s disease (AD) and Parkinson’s disease (PD). These diseases are age-associated and most often have a late debut of the manifestation with a subsequent stage of progression leading to signs of dementia with similar symptoms: memory impairment, orientation problems, difficulties in performing service functions, etc. AD and PD are referred to as “protein misfolding” diseases because deposits of improperly-folded modified proteins are detected in specific areas of the patient brain [1,2,3]. In the case of AD, these deposits contain β-amyloid proteins and hyperphosphorylated tau protein, which, respectively, form extracellular plaques and intracellular fibrillar tangles [4]. In contrast, for PD the deposits—called Lewy bodies—are formed due to the accumulation of α-sinuclein protein in dopaminergic neurons mainly of the substantia nigra, as well as in other regions of the brain [5]. In both AD and PD, neurodegeneration processes are generally accompanied by neuroinflammation [6].

At the same time, AD and PD have different pathogenetic mechanisms, which are evinced in different manifestations of the diseases and are reflected in differences in the methods of their treatment [7]. The pathogenesis of PD is considered as a result of the reduction of dopaminergic activity of neurons of the substantia nigra, which leads to defects in movement control associated with muscle rigidity and tremor at rest and coordination disorders [8]. AD is characterized by the death of neurons and the loss of synaptic transmission in the brain regions responsible for learning and memory (cerebral cortex, temporal and parietal lobes and parts of the frontal cortex and cingulate gyrus), which is the cause of the appearance of cognitive disorders [9]. Despite some overlap of clinical symptoms of the “protein misfolding” diseases, the different mechanisms of AD and PD pathogenesis account for distinctions between course of the diseases: the disturbance of motor functions in PD in most cases does not leads to dementia; and conversely, in AD, mental disorders are not always accompanied by impaired motor activity and coordination [10]. The effectiveness of the treatment of these diseases depends strongly on how early the diagnosis was made and when the specific therapy for AD and PD was started [11]. This is why great importance is attached to the search for associations of neurophysiological signs of the disease development with other indicators of functioning of the organism (biochemical, cytological and immunological), which could be used as specific markers for diagnosis and prognosis of the course AD and PD.

## 2. Inflammation as A Main Immune Process Associated with AD and PD

For both diseases, the process of chronic inflammation in the brain (neuroinflammation) is characterized. Neuroinflammation plays a central role in the development of PD and AD [6]. This process involves not only resident cells (microglia, astrocytes, neurons) of the central nervous system (CNS) but also the cells and humoral factors of the peripheral immune system that penetrate into the brain [12,13,14,15,16]. To date, there is no definite answer to the question whether neuroinflammation is the result or the cause of the development of the neurodegenerative disorders [17]. At the same time the latter assertion is supported by multiple studies indicating that activated microglia, being a source of pro-inflammatory and oxidative mediators with a neurotoxic effect, contributes to the aggravation of inflammation, neurodegeneration and nerve tissue dysfunction. Along with this, it is well known that the first signs of AD and PD are most often manifested in old age, in which the immune system is characterized generally by a state of chronic inflammation, so-called “inflammaging”. It has been shown that this status is manifested, in particular, by the age-related increase in pro-inflammatory mediators in peripheral blood [18]. This was the basis for the assumption that peripheral inflammatory processes can stimulate the development of neuroinflammation and neurodegeneration [19,20,21,22,23]. Therefore, a number of authors suggested that the influence of the peripheral immune system on the process of neuroinflammation can occur due to the changes in the cytokine network [24,25]. This concept allows us to consider the characteristics of immune status, obtained by the analysis of peripheral blood of patients, as informative indicators for clinical diagnostics of the AD and PD development and for the option of immunotherapeutic approaches to the therapy of these diseases.

Nevertheless, the problem of the cause-effect relations between regional and systemic inflammatory processes in the development of PD and AD remains open. On the one hand, it has been shown recently that peripheral immune response can influence regional inflammation in the brain and exacerbate neurodegenerative processes [26,27,28,29,30]. It has also been demonstrated that proinflammatory mediators induced during activation of the innate and adaptive immunity can penetrate through the blood–brain barrier and affect the CNS, contributing to an exacerbation of neurodegeneration by activation of primed microglial cells [30,31]. The possibility of overcoming this barrier is characteristic not only of humoral factors but also of immune cells that infiltrate the sites of inflammation in the brain [13,24,32,33]. On the other hand, there is evidence of the effect of regional neuroinflammation on peripheral immune processes. In particular, it has been shown that the progression of neurodegradation during PD leads to a significant increase in the level of circulating α-sinuclein protein in the blood and this protein causes an essential systemic inflammatory response [34,35].

A series of studies is devoted to the αgenetic associations between inflammatory factors and AD or PD with the aim to define genetic determinants regulating immune inflammatory response. Common genetic changes associated with the risk of these diseases development have not been identified yet [36]. Loss-of-function variants of genes were described as risk factors for AD, among them TREM2, the triggering activating receptor expressed on myeloid cells and CD33 linked to reduced β-amyloid protein phagocytosis by microglia [37,38]. Several variants of *TREM2* exon 2 were presented only in AD cases and showed highly significant association with an increase in AD risk. Such effect of mutations in *TREM2* is believed to be mediated by disturbance of immune response initiation in macrophages and dendritic cells and of phagocytosis control in microglia, which could be relevant to the clearance of β-amyloid proteins [39].

The brains of individuals with PD show up-regulation of major histocompatibility complex class II (MHC-II) antigens, suggesting the involvement of HLA-DR-positive microglia in pro-inflammatory process [40,41]. A genome-wide association study (GWAS) allowed to detect a novel association of sporadic and late-onset of PD with the HLA region [42]. GWAS also provided a study of association of single nucleotide polymorphisms (SNPs) with PD. It was found that increased expression of seven HLA genes (*HLA-B*, *HLA-C*, *HLA-DQA1*, *HLA-DQB1*, *HLA-DQB1-AS1*, *HLA-DRB1* and *HLA-DRB5*) and decreased expression of four genes (*HLA-DOB*, *HLA-DQA2*, *HLA-DQB2* and *HLA-DRB6*) is associated with the risk of PD [43]. Furthermore, it was demonstrated that PD is associated with both structural and regulatory elements in HLA genes [44]. It was also shown that MHC-II expression is required for α-synuclein-induced activation of microglia and genetic polymorphism of HLA alleles associated with the risk of prolonged neuroinflammation [45]. Altogether these findings emphasize the role of inflammatory reactions in PD pathogenesis. No conclusive association was found until now between MHC-II expression pattern and AD progression.

While a potential role of neuronal MHC-I expression in PD was described [46], there is no enough evidences of a significant risk for MHC-I genes in PD progression. In contrast, it has been suggested that genetic determinants of MHC-I are involved in AD progression. AD association with MHC-I *HLA-A2* allele is widely discussed in a number of studies but results of the studies are discordant. In one way, an association between the *HLA-A2* allele and AD was described by several authors [47,48]. However, this association was disproved in other studies [49,50,51]. Such inconsistency of conclusions might result from clinical or genetic heterogeneity of the populations and frequency of *HLA-A2* allele between patients. A meta-analysis of AD cases and control studies before 2014 year that evaluated a relationship between *HLA-A* and AD supports that *HLA-A2* showed to be a mild risk factor of AD with significant results only in some populations [52]. Nevertheless, this association may indicate an involvement of neuronal MHC-I in neuroinflammatory processes and immune-mediated neurodegeneration, suggesting a role T cell response in AD aetiology [53]. It should be noted that neuronal MHC-I expression was also described to be linked to modulation of synaptic function in hippocampal and cortical areas [54,55].

## 3. Alterations of Peripheral Cytokine Profiles in PD and AD

As it is noted above, the process of neuroinflammation accompanying PD and AD is associated with alterations in the peripheral immune system, including the cytokine network. However, the published studies contain contradictory data concerning changes in cytokine production in patients with AD and PD. In particular, an essential increase of serum level of TNFα for patients with PD and AD has been demonstrated by many groups [20,22,56,57,58,59,60,61,62,63]. Nevertheless, some authors claim that there is no significant difference in the serum TNFα between the control group and the AD group [64,65]. A significant increase of serum IL-1β has been also considered as a biomarker for the diseases [20,56,57,59,62,66,67] but a number of investigations testify against the difference in serum IL-1β between patients with PD or AD and healthy donors [61,65]. There are similar contradictions in the data on the alteration of the serum level of IL-1α in PD and AD that is decreased for the patients [66], or is unchanged in AD [22,65]. A similar situation is observed for the data on the disease-related alterations of IL-8 and IFNγ. The published results report both increased [58,65] and not altered [22,59] levels of the cytokines in patients with AD. No significant differences for these cytokines were demonstrated between patients with PD and healthy donors [62]. Concerning IL-18, the majority of publications indicate an increase in serum level of the cytokine for AD [59,68,69], although there are reports showing no alterations in this level [70,71]. In contrast to AD there is a lack of data about IL-18 serum level alteration in PD. The serum level of IL-12 in patients with AD is increased as distinct from patients with PD [59,61] and, vice versa, the registered level of IL-2 and C-reactive protein in the blood of PD patients is higher compared to healthy donors, whereas in AD these cytokines are not significantly changed [22,59,60,62,65,72].

The contradictions in the data mentioned above on the levels of cytokines in the blood can be associated with different stages of the clinical course of the diseases. There is much concern about age-matched healthy controls for studies of age-related diseases. Thus, the very large heterogeneity of the immune statement of aged volunteers can also explain the contradictions in reported results. Additionally, it is known that there are essential variations in the results of the experiments performed with different approaches and different commercial kits.

Nevertheless, despite a wide scatter of data, an overall current representation of similarities and differences in serum levels of the different measureable cytokines between patients with AD and PD can be found using meta-analysis of a number of related publications. Such a type of study using 40 published works has demonstrated that AD is characterized by increased serum levels of IL-6, TNFα, IL-1β, TGFβ, IL-12 and IL-18 [59]. For PD, the meta-analysis of 25 related studies determined higher peripheral concentrations of IL-6, TNFα, IL-1β, IL-2, IL-10, C-reactive protein and RANTES [62]. Some cytokines seem to show an elevated serum level in either AD or PD alone. For AD, these are IL-12, IL-18 and IFNγ—cytokines, known to stimulate Th1 differentiation, lymphocytes adhesion, migration and cytotoxity, MHC-I and MHC-II expression [73,74]. PD is associated with elevated levels of C-reactive protein, IL-2 and IL-10, known to regulate complement system activation, suppress Th1 differentiation and decrease MHC-II expression [75,76,77,78] (Table S1). The clinical significance and pathological role of the elevated cytokine levels remains a subject of debate.

In contrast, the levels of IL-6, IL-1β and TNFα in the blood appear to be elevated in patients with both AD and PD, which is the evidence of systemic inflammation that accompanies both of these neurodegenerative diseases. Interestingly, there was an evidence of the ability of IL-6 to penetrate the blood-brain barrier, as well as the involvement of this cytokine in memory consolidation [79]. It is possible that an increase in the production of this cytokine, having both pro- and anti-inflammatory properties, is a protective reaction of the peripheral immune system. The pro-inflammatory cytokines IL-1β and TNFα is also known to modulate the statements of neurons. It has been demonstrated that these cytokines exert variable (inhibiting or supporting) synapse-specific effects on long-term potentiation (LTP; a persistent increase in synaptic strength required, in particular, for memory and learning) maintenance [80,81,82]. It was also shown that IL-1β and TNFα in combination with IFNγ can exacerbate the pathology in AD due to alterations of the β-amyloid precursor protein (βAPP) metabolism resulting in triggering the production of β-amyloid peptides [83,84].

To conclude, establishing of peripheral cytokine applications as biomarkers of PD and AD is complicated by the essential individual differences in cytokine levels among the patients. Nevertheless, presumably, the use of a combined analysis of a number of peripheral cytokines may find in future a diagnostic application in PD and AD.

## 4. The Role of Oxidative Stress in PD and AD: Products of Oxidative Stress in the Peripheral Blood as Biomarkers of PD and AD

Oxidative stress is considered as one of the main factors in the pathogenesis of neurodegenerative diseases. An increased concentration of free radicals in conjunction with a decrease in antioxidant protection leads to damage of intracellular proteins, lipids and DNA in the nerve tissue [85,86,87,88]. Recently, it has been shown that the mitochondrial stress-induced accumulation of the oxidized form of dopamine in human neurons is one of the key processes for PD development [89]. In the group of patients with PD, an increase in the number and activity of mitochondria in neutrophils was revealed [90]. Furthermore, it was demonstrated a possibility of application of mitochondria-targeted antioxidants for treatment of PD [91]. A causative role of mitochondrial dysfunction in the brain in the pathogenesis of AD is also discussed [92]. In particular, an elevated level of oxidative stress markers was revealed in mitochondria, isolated from peripheral lymphocytes of AD patients [93].

Along with the neurodegenerative effect of oxidative stress in nerve tissues, it should not be excluded that neurodegeneration in AD itself can provoke intensification of reactive oxygen species (ROS) production [94]. Activation of microglia in PD triggers increased levels of pro-inflammatory mediators (TNFα, IL-1β and IL-6) and ROS, which aggravates microglia-derived inflammation and neurodegeneration [95].

It has been also shown that, in PD and AD, the balance of antioxidant and oxidant system activity is disturbed in different cells. AD patients are characterized by significant increases of the oxidized form of RNA 8-hydroxyguanosine (8OHG) in neurons and an 8OHG level that is inversely correlated with the progression of the disease [96]. Progression of neurodegeneration in PD is also accompanied by accumulation of ROS, as well as by oxidative damage and violation of antioxidant protection, which can be detected not only in brain cells but also in peripheral immune cells and serum of the patients. For example, in peripheral blood mononuclear cells (PBMC) from the patients with untreated PD, an increase in the ROS level was demonstrated [97]. In addition, in the PD group of patients, the marker of induced genomic damage, the 8-hydroxy-2′-deoxyguanosine (8-OHdG) in leukocytes, as well as the product of lipid peroxidation malondialdehyde (MDA) in the blood plasma was increased concurrently with the reduced level of antioxidant protection [98,99].

Alterations of some biochemical and immune characteristics found in patients with neurodegenerative disease progression may underlie the consideration of the characteristics and their combinations among potential peripheral biomarkers of both AD and PD. For PD and AD, a number of such markers include, in particular, the above-mentioned MDA—a product of lipid peroxidation [86,100]. Products of oxidative stress in patient blood can also be referred to the indicators of the development of AD. For instance, the level of 8-OHdG in plasma and in peripheral lymphocytes in the AD group were significantly higher compared to the control group and it was observed together with a considerable decrease in various components of anti-oxidative protection in the blood [101,102,103]. The effect of oxidative stress in AD is manifested by high levels of oxidized proteins, the products of lipid peroxidation and by the toxic species of ROS and oxidative modifications in nuclear and mitochondrial DNA. In particular, a significant increase in the degree of lipoprotein oxidation was observed in the peripheral blood of AD patients [104].

Neutrophils are the main source of ROS production in the sites of inflammation. Therefore, these cells could play a role in the development of neurodegeneration. Changes in the functional characteristics of neutrophils in patients with neurodegenerative diseases have still been poorly studied. However, it was shown that the activity of NO-synthase in neutrophils (nNOS) from the peripheral blood of patients with PD was increased, resulting in an elevated production of nitrogen monoxide (NO). At the same time the activity of the antioxidant enzyme catalase was significantly lower in the neutrophils of PD patients compared to healthy donors [105]. In addition, it has been demonstrated that another protective function of neutrophils—phagocytosis—is decreased in PD patients [106].

A possible participation of neutrophils in the development of AD has been demonstrated [107]. In a mouse model of AD, it has been shown that a recombinant form of β-amyloid protein Aβ42 promotes an increase in the adhesion of neutrophils and their migration through the epithelial barriers. Additionally, both neutrophil depletion and suppression of the activity of the adhesion molecule LFA-1 led to a decrease in neuropathology and to memory recovery in mice with developed cognitive dysfunction [108].

## 5. HSP70 as A Possible Biomarker for Neurodegenerative Diseases

Among the potential peripheral biomarkers of neurodegenerative diseases, the studies in which these indicators are searched by gene expression analysis in samples of peripheral blood cells of the patients are worthy of special attention. In particular, it was shown that in PD, alterations of gene expression in cells from the sources of neurodegeneration and from peripheral blood, had a largely similar pattern [109]. Peripheral blood cells also showed significant changes in gene expression already at an early stage of the development of the disease, which differed for PD and AD [110,111]. It was found that the gene encoding the chaperone protein HSP70 was among five genes considered as optimal predictors of PD [110]. This is not surprising, because AD and PD are referred to as “protein misfolding” diseases and one of the factors underlying their pathogenesis is reduced activity of the protein homeostasis system, leading to accumulation of neurotoxic aggregates of the modified proteins in the cells of the nervous tissue. Neuroprotective effects of HSP70 were demonstrated in several different models of nervous system injury using transgenic animals overexpressing this protein [112,113,114,115,116,117,118]. From this point of view, the abnormalities of chaperone-associated system, in particular the HSP70 subfamily, which supports protein homeostasis and cell viability can be considered as one of the key indicators reflecting the development of protein-misfolding diseases [119].

HSP70, a major member of the heat shock protein family, providing correct folding, refolding, disaggregation of protein molecules and participating in the mechanism of chaperone-mediated autophagy aimed at elimination of damaged and aggregated substrates, are among the main components of the protein homeostasis system [120,121]. The possibility of using HSP70 as a peripheral biomarker of neurodegenerative diseases is also evidenced by data demonstrating the changes in the expression of these proteins not only at the gene level but also at the level of the intracellular content of HSP70 in peripheral blood leukocytes of patients with PD [122,123].

Along with intracellular HSP70, the extracellular serum pool of these proteins circulating in the body is also of undoubted interest in the search for peripheral biomarkers of process of neuroinflammation and neurodegenerative diseases, in particular PD and AD [124,125]. It was demonstrated that extracellular HSP70 exhibits potent immunomodulatory effects on innate and acquired immunity [126,127]. At present, there is no reliable evidence that the clinical course of the neurodegenerative diseases is correlated with the level of the serum HSP70 in the peripheral blood of patients but there are numerous data on the considerable alterations of this level for a wide range of pathologies [125,128]. In addition, a positive relationship has been found between the serum level of HSP70 and some markers of inflammation in the elderly, which confirms the involvement of these proteins in the diseases associated with processes of inflammaging [129]. Additionally, age-related differences in the relationship between the expression of HSP70 and the production of reactive oxygen species in the population of human peripheral blood neutrophils, involved in inflammaging, have been demonstrated [130]. Taking into account that the overwhelming number of neurodegenerative diseases is observed in the population of elderly people, it can be assumed that analysis of the level of intracellular and extracellular pools of HSP70 in peripheral blood samples of the patients is a promising approach for studying the mechanisms of the pathogenesis of PD and AD.

## 6. Conclusions

The recent studies presented in the mini-review show an increased attention focused on the involvement of immune processes in the pathogenesis of the neurodegenerative diseases, in particular, AD and PD. The research efforts are also aimed at the search of diagnostically important biomarkers involved in peripheral immune reactions accompanying the processes of neurodegeneration. In this mini-review, we emphasize the comparison of the relationships for the most common age-associated neurodegenerative diseases—AD and PD, which related to the processes of regional (neuroinflammation) and system (inflammaging) inflammation. The specified comparative analysis was aimed at identifying common patterns of bi-directional interaction of the CNS and peripheral immune system, characteristic of neurodegenerative diseases.

The accumulated literature data do not raise doubts that interactions of regional and peripheral chronic inflammatory processes, is largely associated with characteristic for neuroinflammation abnormal blood-brain barrier permeability for soluble factors and circulating cells of the immune system. Nevertheless, the problem of the causal relationship between regional and system inflammatory processes in the development of PD and AD remains open. Analysis of the gene-dependent associations of the immune system with the risk of PD and AD development has not revealed significant evidence of such associations common for both diseases. General genetic disorders/changes associated with the risk of developing these diseases have not yet been detected, although several different genes related to the immune system was described as the risk factors for AD and PD. A number of studies have demonstrated that process of neuroinflammation accompanying PD and AD is associated with alterations in the peripheral immune system cytokine network. Elevated levels of IL-6, IL-1β and TNFα often found in the blood of both AD and PD patients can be considered as the evidence of systemic inflammation accompanied both of these neurodegenerative diseases. Oxidative stress is believed as one of the main factors in the pathogenesis of neurodegenerative diseases. It has also been shown that in PD and AD the balance of antioxidant and oxidant system activity is disturbed in different cells. With respect to the discussion on peripheral biomarkers of neurodegenerative diseases, studies in which these indicators are searched by gene expression analysis in samples of peripheral blood cells of the patients are worthy of special attention. It was found that the gene encoding the chaperone protein Hsp70 was among five genes considered as the predictors of PD. Along with intracellular HSP70, the extracellular serum pool of these proteins circulating in the body is also of undoubted interest in the search for peripheral biomarkers of process of neuroinflammation and neurodegenerative diseases, in particular PD and AD. Taking into account that the overwhelming number of neurodegenerative diseases is observed in the population of elderly people, it can be assumed that the analysis of the level of intracellular and extracellular pools of HSP70 in peripheral blood samples of the patients is a promising approach for studying the mechanisms of the pathogenesis of PD and AD.

## Supplementary Materials

Supplementary materials can be found at www.mdpi.com/1422-0067/18/12/2633/s1.
