# Supplementary material for: Similarity and Differences in Inflammation-Related Characteristics of the Peripheral Immune System of Patients with Parkinson’s and Alzheimer’s Diseases
Source: Int J Mol Sci. 2017 Dec 6;18(12):2633. doi: 10.3390/ijms18122633 (PMC5751236; doi:10.3390/ijms18122633)
Supplement: Supplementary file 1 [file ijms-18-02633-s001.pdf]

**Alzheimer's disease**		**Parkinson's disease**	
IL-12	cytotoxic, antiangiogenic, ↑CD8$^+$ CTL, ↑NK cells, ↑Th1, ↑IFNγ, ↑TNFα	IL-10	↑B cells, ↓Th1, ↓MHC-II, ↓TNFα, ↓IL-1β, ↓IL-12, ↓IFNγ
IL-18	↓IgE & IgG1, ↑IgG2a, ↑IFNγ	IL-2	↓ IL-15, ↑IL-12
IFNγ	↑MHC-I & MHC-II, ↑Th1, ↓Th2, ↑NK cells, ↑IgG2a & IgG3, ↑adhesion & migration of leukocytes	CRP	↑complement system
IL-6	↓TNFα, ↑Th17, ↑IL-1RA, ↑IL-10, BBB crosses , memory consolidation	IL-6	↓TNFα, ↑Th17, ↑IL-1RA, ↑IL-10, BBB crosses, memory consolidation
IL-1β	↑COX2	IL-1β	↑COX2
TNFα	inflammation	TNFα	inflammation

Table S1. Summary of key published results related to the similarities and differences between AD and PD in cytokine expression and their effects on the immune and other processes. Similar effects for AD and PD are allocated in cells filled with grey color.
